# Simple and rapid determination of homozygous transgenic mice via *in vivo* fluorescence imaging

**DOI:** 10.18632/oncotarget.5535

**Published:** 2015-10-12

**Authors:** Xiaolin Lin, Junshuang Jia, Yujuan Qin, Xia Lin, Wei Li, Gaofang Xiao, Yanqing Li, Raoying Xie, Hailu Huang, Lin Zhong, Qinghong Wu, Wanshan Wang, Wenhua Huang, Kaitai Yao, Dong Xiao, Yan Sun

**Affiliations:** ^1^ Cancer Research Institute, Southern Medical University, Guangzhou, China; ^2^ Joint Program in Transfusion Medicine, Children's Hospital Boston, Harvard Medical School, Boston, Massachusetts, USA; ^3^ Institute of Comparative Medicine & Laboratory Animal Center, Southern Medical University, Guangzhou, China; ^4^ Department of Anatomy, Guangdong Provincial Key Laboratory of Construction and Detection in Tissue Engineering, School of Basic Medical Science, Southern Medical University, Guangzhou, China

**Keywords:** transgenic mice, fluorescence reporter gene, *in vivo* qualitative and quantitative fluorescence imaging, homozygote

## Abstract

Setting up breeding programs for transgenic mouse strains require to distinguish homozygous from the heterozygous transgenic animals. The combinational use of the fluorescence reporter transgene and small animal *in-vivo* imaging system might allow us to rapidly and visually determine the transgenic mice homozygous for transgene(s) by the *in vivo* fluorescence imaging. RLG, RCLG or Rm17LG transgenic mice ubiquitously express red fluorescent protein (RFP). To identify homozygous RLG transgenic mice, whole-body fluorescence imaging for all of newborn F2-generation littermates produced by mating of RFP-positive heterozygous transgenic mice (F1-generation) derived from the same transgenic founder was performed. Subsequently, the immediate data analysis of the *in vivo* fluorescence imaging was carried out, which greatly facilitated us to rapidly and readily distinguish RLG transgenic individual(s) with strong fluorescence from the rest of F2-generation littermates, followed by further determining this/these RLG individual(s) showing strong fluorescence to be homozygous, as strongly confirmed by mouse mating. Additionally, homozygous RCLG or Rm17LG transgenic mice were also rapidly and precisely distinguished by the above-mentioned optical approach. This approach allowed us within the shortest time period to obtain 10, 8 and 2 transgenic mice homozygous for RLG, RCLG and Rm17LG transgene, respectively, as verified by mouse mating, indicating the practicality and reliability of this optical method. Taken together, our findings fully demonstrate that the *in vivo* fluorescence imaging offers a visual, rapid and reliable alternative method to the traditional approaches (i.e., mouse mating and real-time quantitative PCR) in identifying homozygous transgenic mice harboring fluorescence reporter transgene under the control of a ubiquitous promoter in the situation mentioned in this study.

## INTRODUCTION

Transgenic mice continue to be heavily useful and powerful tools for dissecting physiological and pathological processes in biomedical research [1–11]. For breeding and maintaining transgenic mouse strains, or for particular experiments, in which gene dosage effects might have a functional impact, it is extremely useful to define a rapid and accurate approach for distinguishing the mice homozygous for the transgene(s) from the heterozygous ones [1]. In transgenic animals produced by pro-nuclear microinjection of DNA [1, 12] and lentivirus-mediated gene delivery [13, 14], transgene inserts into the genome at random sites. Taking this information into account, the real-time quantitative PCR, regarded as an accurate and reliable method to determine zygosity in transgenic mice, has been introduced in transgenic research to overcome several drawbacks, such as time-consuming, tedious, specialized techniques and/or ambiguity in the results, of previously established methods, for example, Southern blot hybridization, dot blot hybridization, fluorescence *in situ* hybridization and mouse mating [15].

The applications of fluorescent proteins for *in vivo* imaging have opened many new areas of research [16]. The important advances in this field have been the development of various transgenic mice expressing various fluorescent proteins, including enhanced green fluorescent protein (EGFP) [14], red fluorescent protein (RFP) [17–19] and cyan fluorescent protein (CFP) [20], etc. The *in vivo* green, red or cyan fluorescent protein imaging performed in the reporter transgenic mice provides a simple, rapid and visual approach to performing genotyping by assaying the reporter gene expression of either whole body (newborn) or tail tip or ear, in replace of PCR-based genotyping.

To make full use of the above-mentioned advantages of the fluorescent imaging in non-invasively and visually characterizing transgenic mice, some general transgenic vectors have been successfully developed (Supplementary Table S1) and applied for developing transgenic animals used in biomedical research [14, 17, 18, 21–25]. These general transgenic vectors harbor the reporter transgene (i.e., EGFP, RFP, etc) under the transcriptional control of a ubiquitous promoter and carry multiple cloning site (MCS), into which target transgene(s) can be inserted (Supplementary Table S1).

In theory, about two-fold differences in the expression level of transgene(s), including reporter gene, between transgenic mice homozygous and heterozygous for the transgene(s) exist, implying that visualizing the reporter gene expression by whole-body or organ-specific quantitative fluorescence imaging should be employed to simply, rapidly and visually distinguish homozygous from heterozygous transgenic animals. Furthermore, at present the *ex vivo* and *in vivo* qualitative and quantitative fluorescence imaging can be readily realized by the small animal *in-vivo* imaging systems, such as the IVIS Lumina Imaging System (Referred to as IVIS system) from Xenogen.

In this study, we will employ our practices for identifying homozygous transgenic mice by this visual approach to fully demonstrate, for the first time, how to simply, rapidly, visually and reliably determine homozygous individuals among the transgenic littermates by *ex vivo* and *in vivo* fluorescence imaging, in replace of the real-time quantitative PCR and traditional mouse mating.

## RESULTS

### Principle for distinguishing homozygous from heterozygous transgenic alleles by *in vivo* fluorescence imaging

**Figure 1:**
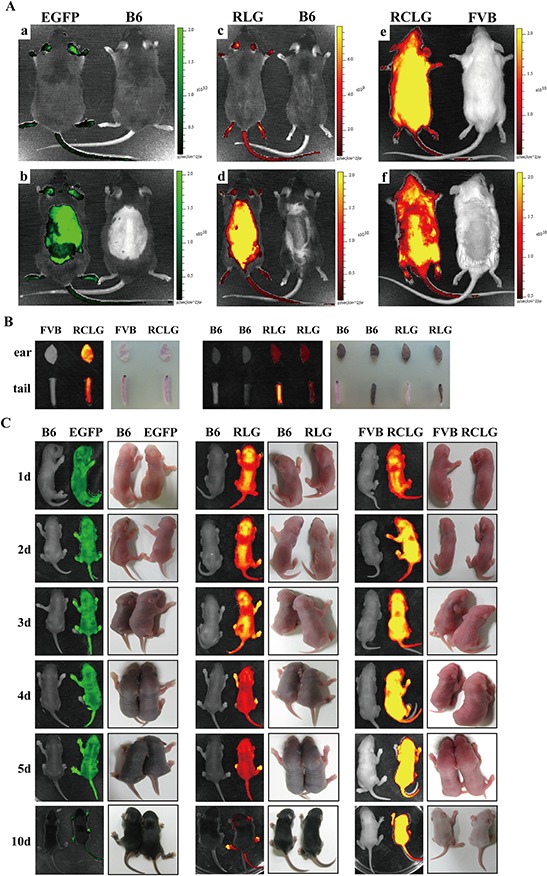
Whole animal and organ fluorescence imaging **A.** Whole-body fluorescence imaging for EGFP, RLG and RCLG transgenic mice. Mice presented in Figure 1A-a, -c and -e were not shaved, whereas mice shown in Figure 1A-b, -d and -f were shaved from the neck to the lower torso. Fluorescent intensity is recorded as photons/sec/cm^2^, and the color of the signal represents the amount of EGFP or mRFP protein present. See the figure legend of Supplementary Figure S1 for details on EGFP transgenic mice. Abbreviation: B6, C57BL/6J; EGFP, EGFP transgenic mice; RLG, RLG transgenic mice; RCLG, RCLG transgenic mice. **B.** Organ-specific fluorescence imaging for mouse ears and tail tips of the adult RLG and RCLG transgenic mice. **C.** Whole-body fluorescence imaging for the EGFP, RLG and RCLG transgenic mice at the age of 1, 2, 3, 4, 5 and 10 days.

Newborn EGFP-Luc double transgenic mice (designated as L2G85) indicated that both bioluminescent and fluorescent signals in heterozygote were significantly lower than those in homozygote [26], while the fluorescence and bioluminescent intensity of the whole body of homozygous and heterozygous L2G85 mice was 6 × 10^9^ and 2 × 10^9^ (fluorescence intensity), and 7 × 10^11^ and 4 × 10^11^ (bioluminescent intensity) [26], respectively. Moreover, compared with heterozygote, the homozygous RFP transgenic mice mentioned in Figure 1 of the paper [27] and the homozygous EGFP transgenic mice (Supplementary Figure S1) displayed more strong fluorescence and very high fluorescence intensity [7.33 × 10^10^ (homozygote) vs 3.725 × 10^10^ (heterozygote)] (Supplementary Figure S1).

In theory, two-fold differences in copy numbers of the transgene between homozygous and heterozygous transgenic mice can result in the two-fold diversities in the expression level of transgene(s) [including the reporter gene(s)] between homozygous and heterozygous transgenic mice. Actually, the *in vivo* and *ex vivo* quantitative fluorescent imaging revealed the following three situations: approximate two-fold differences (Supplementary Figure S1), less than 2-fold differences (Figure 4C, Figure 6G, Supplementary Figure S2, Supplementary Figure S3, Supplementary Figure S4) and more than 2-fold differences [26] in the expression level of the reporter transgene between homozygous and heterozygous transgenic mice, due to the various reasons, such as (1) the different position of each individual of littermates placed in a 10 cm Petri dish when they were imaged together by the small animal *in-vivo* imaging system, (2) imaging angle, (3) body posture of animals, (4) clipped mouse ear size/shape, (5) with/without hair or (6) other unknown causes. Anyway, the transgenic mice homozygous for the transgene EGFP or mRFP indicate the more strong fluorescence, compared with the heterozygous transgenic mice, suggesting that visualizing the reporter gene expression by whole-body or organ-specific fluorescence imaging should be used to simply, rapidly and visually distinguish homozygous from heterozygous transgenic mice, as strongly supported by the following data from this study.

### Whole animal (*in vivo*) and organ (*ex vivo*) fluorescence imaging

Next, which kind of fluorescence imaging fashion styles [whole-body (newborn) VS organ-specific fluorescence imaging] can be selected to easily and rapidly distinguish homozygous from heterozygous transgenic animals?

Whole-body imaging of adult EGFP, RLG and RCLG transgenic mice primarily depends upon coat color. In wild-type FVB mice and RCLG transgenic mice which have white fur, the red fluorescence was only detected on the whole unshaven (Figure 1A–e, left) and shaven (from neck to lower torso) (Figure 1A–f, left) body of RCLG transgenic strains, but not the whole unshaven (Figure 1A–e, right) and shaven (Figure 1A–f, right) body of wild-type FVB mice under certain exposure time, suggesting that the detectable red fluorescence emitted from the whole unshaven and shaven body of RCLG transgenic strains is not autofluorescence. Additionally, the total fluorescence intensity of the entire unshaven body of RCLG transgenic mouse (Figure 1A–e, left) is bigger than that of the whole shaven body of RCLG transgenic mouse (Figure 1A–f, left), indicating that the shaven fur took some fluorescence.

This is in contrast to wild-type C57BL/6J (B6), EGFP and RLG transgenic mice which have a black coat color. In these animals, the black fur does not autofluoresce but rather absorbs light. Therefore, neither the unshaved (Figure 1A–a, c, right) or shaved (Figure 1A–b, d, right) parts of the wild-type animal yielded fluorescent signals. In EGFP and RLG transgenic mice, only those areas that have been shaved or those areas (i.e., eyes, ears, legs and tail) that have little or no fur yielded fluorescent signals (Figure 1A–a, b, c, d, left).

In addition, organ fluorescence imaging of mouse ear and tail tip from RLG and RCLG transgenic mice (3-week-old) indicates that mouse ears and white tail tips from RLG and RCLG transgenic mice do not interfere in imaging (Figure 1B), whereas black tail tip from RLG transgenic mice does interfere in imaging (Figure 1B).

Whole-body imaging of EGFP, RLG and RCLG transgenic mice on postnatal day 1, 2, 3, 4, 5 and 10 is shown in Figure 1C. Whole-body imaging of EGFP and RLG transgenic mice (C57 genetic background) (1 to 3 days old) and RCLG transgenic mice (FVB genetic background) (1 to 10 days old) does not completely depend upon coat color (Figure 1C) because these transgenic mice at this period have no fur and pigment deposition (Figure 1C), but when EGFP and RLG transgenic mice gradually grow up, the black pigment in skin and black fur seriously interfere in whole-body imaging (Figure 1C). Moreover, it is very easy to perform whole-body imaging for newborn mice (1–3 days old) as they would not like to move.

Therefore, to avoid fur interference, conveniently perform the whole-body fluorescence imaging for all of newborn F2-generation littermates and take the accessibility of mouse organs into account, the newborn transgenic mice (1 to 5 days old) and cropped ear are firstly chosen to perform *in vivo* and *ex vivo* qualitative and quantitative fluorescence imaging to identify homozygous transgenic mice.

### Generation of RLG transgenic mice

**Figure 2:**
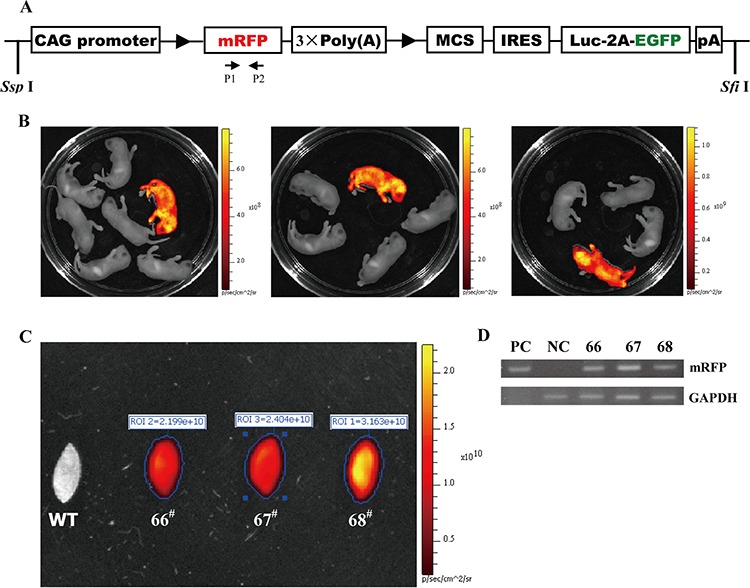
Three transgenic founders derived from the microinjection of CAG-RLG transgenic construct into one cell-stage fertilized embryos **A.** Schematic diagram of CAG-RLG transgenic construct used to generate the RLG transgenic mice. The primer pair P1/P2 represented by small arrows are used in PCR analysis of genotype to detect the reporter transgene mRFP. The construct map is not drawn to the scale. Abbreviations: CAG promoter: CMV early enhancer/chicken β actin promoter; mRFP: monomeric red fluorescent protein; Luc: firefly luciferase; EGFP: enhanced green fluorescent protein; pA: polyadenylation signal; ► : *lox P* site. **B.** Screening the RLG transgenic founders by the *in vivo* fluorescence imaging. Three foster mothers gave birth to six, five and eight F0 pups, respectively. Three mRFP-positive RLG transgenic mice (referred to as 66^#^, 67^#^ and 68^#^) were found 2–3 days after birth via the mRFP assay by using the IVIS system. **C.** Detecting mRFP expression in mouse ears of the adult RLG transgenic mice. When mRFP expression was tested by the *ex vivo* quantitative fluorescence imaging, the ear from adult 68^#^ showed more strong red fluorescence (photon signal:3.163 × 10^10^), and the ears from adult 66^#^ and 67^#^ displayed the moderate fluorescence signals (photon signal: 2.199 × 10^10^ and 2.404 × 10^10^, respectively). The quantified photonsignal (photon/sec) of each ear was shown above each image. WT: wild-type mouse. **D.** mRFP-positive mice verified for the RLG transgene presence by PCR analysis. Three mRFP-positive mice (i.e., 66#, 67# and 68#) were individually analyzed by PCR for the genomic integration of transgene with tail biopsy-derived DNA from mRFP-positive mice (66^#^, 67^#^ and 68^#^). PCR products were amplified by the primer pair P1/P2 (specific for mRFP) shown in Figure 2A. Lane M1: DL2000 (TaKaRa); lane PC: positive control (pCAG-RLG as template); lane NC: negative control using genomic DNA from WT mouse as template. Data are representative of three independent PCR experiments that yield similar results.

The RLG construct used for microinjection is illustrated in Figure 2A. mRFP expression allows easy identification of RLG transgenic mice by using small animal *in-vivo* imaging system or under stereo fluorescence microscope. Of the 110 embryos transferred to the recipient females, 18 embryos developed to term. Three individuals of 18 siblings are transgenic, as demonstrated by the red fluorescence in the whole body of newborn mice (Figure 2B) and the mouse ear of adult mice (Figure 2C), as confirmed by PCR analysis (Figure 2D). Therefore, three founder animals (referred to as 66^#^, 67^#^and 68^#^) are attained.

### Screening RLG homozygous transgenic mice by *in vivo* and *ex vivo* fluorescence imaging

**Figure 3:**
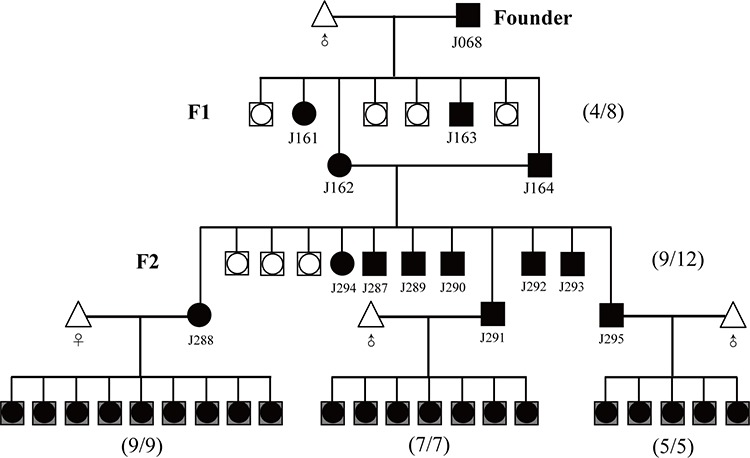
Strategy for distinguishing the homozygous from the heterozygous transgenic alleles by this optical approach Founder (J068, female) was bred to the male partner of wild-type C57 mouse. Littermates were screened for the presence of the transgene RLG by mRFP assay. Numbers in parentheses shown on the right of map imply the ration of transgenic to non-transgenic plus transgenic offspring. ○, male; □, female; ●, transgenic male; ■, transgenic female; △, wild-type C57 mouse; 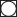
: these mRFP-negative newborn offspring were sacrificed after mRFP assay, so it is impossible to distinguish between male and female. 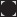
: these mRFP-positive newborn offspring were sacrificed after mRFP assay, so it is impossible to distinguish between male and female.

**Figure 4:**
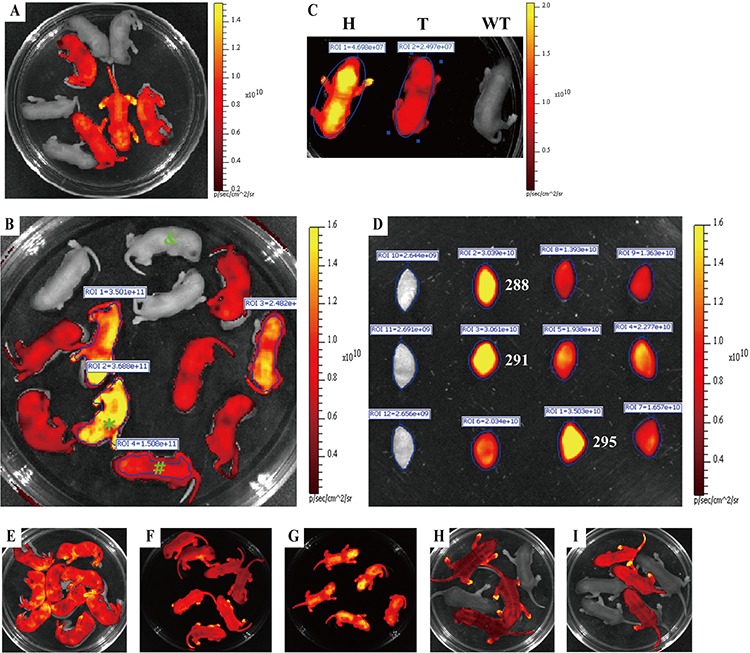
Rapidly distinguishing the homozygous from the heterozygous transgenic alleles by *ex vivo* and *in vivo* fluorescence imaging Strategy for distinguishing the homozygous from the heterozygous transgenic alleles by this visual method is fully demonstrated in Figure 3. mRFP expression of either whole body or mouse ear was assayed by IVIS system. **A.** Newborn offspring (3 day-old) derived from mating between RLG transgenic mouse (J068, ♀) and wildtype C57BL/6J mouse (♂). **B.** Red fluorescence intensity of newborn offspring (3 day-old) derived from mating between J164 (♀) and J162 (♂) RLG transgenic mice. **C.** Whole-body imaging (newborn) of one homozygote (H) [marked by asterisk (*) in Figure 4B], one heterozygote (T) [marked by pound sign (#) in Figure 4B] and mRFP-negative mouse (WT) (marked by “&” in Figure 4B). **D.** Red fluorescence intensity of mouse ears of adult offspring mentioned in Figure 4B. **E–G.** Confirming potential homozygous RLG transgenic mice by mouse mating. The homozygous RLG transgenic mice (i.e., J288, J291 and J295) screened by this optical approach were further confirmed by mating J288, J291 or J295 with wild-type C57BL/6J mice, respectively. Figure 4E, Figure 4F and Figure 4G demonstrate the one typical whole-body red fluorescence imaging for newborn offspring (3 day-old) derived from mating J288 (Figure 4E), J291(Figure 4F) or J295 (Figure 4G) with wild-type C57BL/6J mice, respectively. The results obtained by whole-body fluorescence imaging for newborn offspring derived from mating J288, J291 or J295 with more wild-type C57BL/6J mice, respectively, are summarized in Supplementary Table S3. **H–I.** Confirming the potential heterozygous RLG transgenic mouse by mouse mating. The heterozygous RLG transgenic mice (i.e., J287 and J293) selected by this optical approach were further verified by mating J287 or J293 with non-transgenic partners, respectively. Figure 4H and Figure 4I demonstrate the one representative whole-body red fluorescence imaging for newborn offspring (3 day-old) derived from mating J287 (Figure 4H) or J293 (Figure 4I) with non-transgenic partners, respectively.

**Figure 5:**
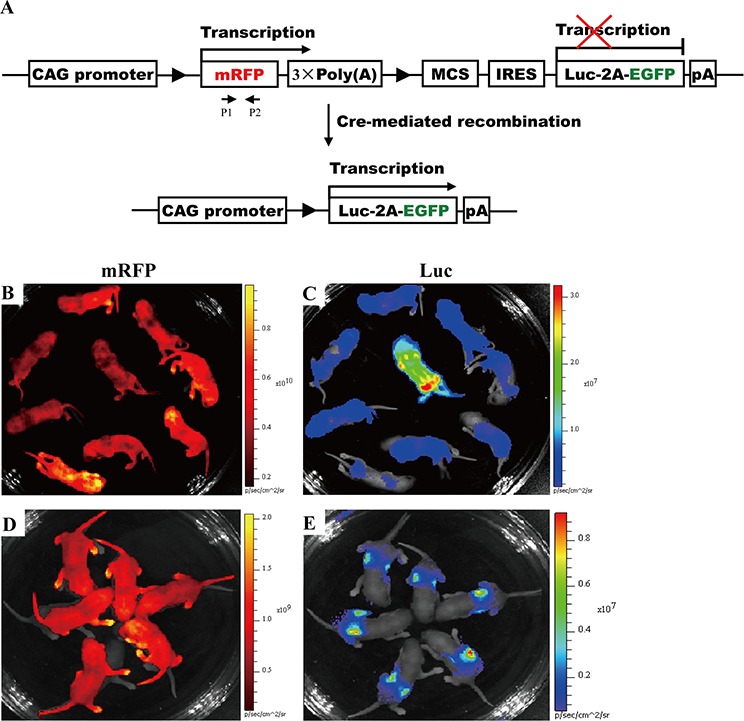
Activation of Luc expression in all of newborn offspring from mating J291 with homozygous EIIa-Cre or Alb-Cre mice **A.** Conditional expression of mRFP and Luc mediated by Cre/*lox* P system. In the absence of Cre-mediated recombination, only mRFP will be transcribed, whereas Luc gene expression is prevented by STOP sequence flanked by *lox* P sites. When Cre-mediated recombination occurs, the floxed mRFP + 3 × PolyA is excised, and Luc expression is activated in a diffuse pattern in RLG/EIIa-Cre double transgenic mice (Figure 5C) or in a liver-restricted pattern in RLG/Alb-Cre double transgenic mice (Figure 5E). Other details as in Figure 2A. **B–C.** Activation of Luc expression in a diffuse pattern in all of newborn RLG/EIIa-Cre mice. As the efficiency of Cre-mediated recombination in RLG/EIIa-Cre mice was not very high, some cells in different organs of RLG/EIIa-Cre mice still harbored mRFP gene. Therefore, mRFP expression was still detectable in whole-body (Figure 5B) and various organs (data not shown) of RLG/EIIa-Cre mice. **D–E.** Activation of Luc expression in a liver-restricted pattern in all of newborn RLG/Alb-Cre mice.

Founder 68^#^ selected from the aforementioned three founder animals (Figure 2) is employed to fully demonstrate how to simply, rapidly and visually distinguish homozygous from heterozygous animals by whole-body and organ-specific qualitative and quantitative fluorescence imaging. Procedure for establishing homozygous RLG transgenic mouse colony by mating heterozygous males and females from founder line 68^#^ is detailedly demonstrated in Figure 3. Brother sister mating of mRFP-positive heterozygous animals (Figure 4A) shows the transgene transmission to the offspring (9 mRFP-positive and 3 mRFP-negative) following expected Mendelian laws (Figure 4B, 4D). The imaging data greatly facilitate us to rapidly and readily find 9 mRFP-positive mice out of 12 littermates. Among 9 mRFP-positive mice, three mRFP-positive transgenic mice [referred to as J288 (♀), J291 (♂) and J295 (♂)] indicate more strong red fluorescence (Figure 4B, 4D) and very high fluorescence intensity (FI) in mouse ears (Figure 4B, 4D and Supplementary Table S2), compared with the rest of 6 mRFP-positive littermates. Moreover, the fluorescence intensity of the whole body of one suspected homozygote and one suspected heterozygote (newborn) is 4.699 × 10^7^ and 2.497 × 10^7^(Figure 4C). Thus, we think that three mRFP-positive transgenic mice [i.e., J288 (♀), J291 (♂) and J295 (♂)] are regarded as homozygous for RLG transgene based on the qualitative and quantitative imaging data, as strongly supported by below-mentioned mouse mating (Figure 4E, 4F, 4G, Figure 5B, 5C, 5D, 5E and Supplementary Table S3). Additionally, this optical approach greatly allows us to easily and rapidly obtain other 7 homozygous RLG transgenic mice [derived from RLG transgenic founders (i.e., 66^#^, 67^#^and 68^#^)] (data not shown), which was strongly verified by mouse mating (data not shown).

### Verifying the above-mentioned homozygous RLG transgenic mice by mouse mating

The results obtained by this optical approach were also confirmed by mating homozygous RLG mice [J288 (♀), J291 (♂) and J295 (♂)] and heterozygous RLG mice [J293 (♀) and J287 (♂)] with non-transgenic partners (Figure 4E–4I and Supplementary Table S3), respectively. RLG transgene transmission follows the expected Mendelian inheritance laws since 100% of homozygous descendants and a fraction of heterozygous offspring show mRFP fluorescence (Figure 4E–4I and Supplementary Table S3). Moreover, the homozygous RLG mouse (i.e., J291), as defined by this optical approach, was crossed to the homozygous EIIa-Cre mice [in which Cre is under the control of a zygotically expressed (EIIa-Cre) promoter] or the homozygous Alb-Cre mice [in which Cre is under the control of a liver-specific albumin promoter] to produce RLG/EIIa-Cre or RLG/Alb-Cre double transgenic mice, respectively. Luc expression was activated in a diffuse pattern in all individuals of newborn RLG/EIIa-Cre mice and (Figure 5C), and in a liver-restricted pattern in all individuals of newborn RLG/Alb-Cre mice (Figure 5E). The above-mentioned findings fully demonstrate the reliability of this optical approach in distinguishing the transgenic mice homozygous for RLG transgene from the heterozygous ones.

Furthermore, these versified homozygous RLG transgenic mice, such as J288 (♀), J291 (♂) and J295 (♂), are subsequently maintained by brother sister mating. The homozygous RLG transgenic mice display no overt phenotype.

### Utility of homozygote identification by this visual method

**Figure 6:**
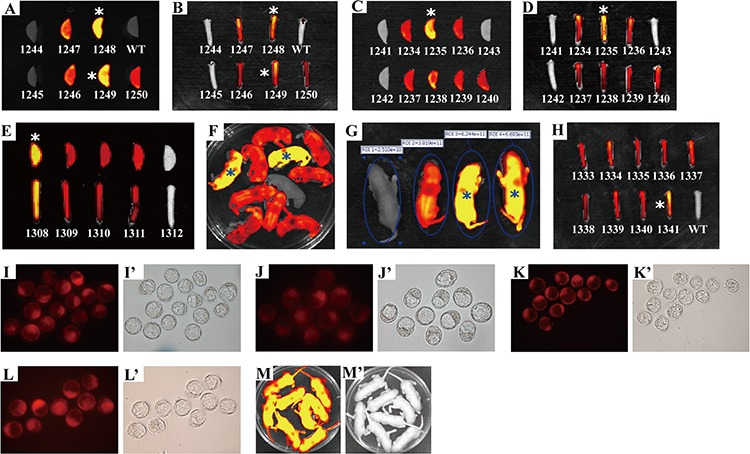
Rapidly and readily distinguishing the homozygous from the heterozygous RCLG transgenic alleles by this visual method Homozygous animals of RCLG transgenic mouse lines were obtained by intercrosses of heterozygotes (Supplementary Table S2) derived from mating between RCLG transgenic founder (190, ♀) and wild-type FVB/N mouse (♂), followed by optically differentiating homozygous transgenic mice by *ex vivo* (mouse ear and/or tail tip) (Figure 6A–6E, 6H) and *in vivo* (whole-body, newborn) (Figure 6F) qualitative (Figure 6A–6F, 6H) and quantitative (Figure 6G and Supplementary Table S2) fluorescence imaging, which was further confirmed by mouse mating (Figure 6I–6M and Supplementary Table S3). The data on *ex vivo* quantitative fluorescence imaging of mouse ear and/or tail tip shown in Figure 6A–6E, 6H are demonstrated in Supplementary Table S2. In addition, these homozygous animals (i.e., 1235, 1248, 1249, 1308 and 1341) identified by this optical approach are marked by asterisk (*). Other details as in Figure 1, Figure 4, Supplementary Table S2 and Supplementary Table S3. **A–H.** Visually identifying the homozygous RCLG transgenic mice by the *ex vivo* and *in vivo* fluorescence imaging. **A–B.** *Ex vivo* fluorescence imaging of mouse ear (Figure 6A) and tail tip (Figure 6B) from offspring (3-week-old) produced by intercrosses of RCLG heterozygotes (590 × 592) (Supplementary Table S2); **C–D.** *Ex vivo* fluorescence imaging of mouse ear (Figure 6C) and tail tip (Figure 6D) from offspring (3-week-old) obtained by intercrosses of RCLG heterozygotes (589 × 596) (Supplementary Table S2); **E.** *Ex vivo* fluorescence imaging of mouse ear (upper) and tail tip (lower) from offspring (3-week-old) obtained by intercrosses of RCLG heterozygotes (587 × 596) (Supplementary Table S2); **F.** Whole-body fluorescence imaging of newborn offspring (3-day-old) produced by intercrosses of RCLG heterozygotes (588 × 592) (Supplementary Table S2) (Note: one homozygous RCLG transgenic mice, defined by this visual method, suddenly died during the growth); **G.** Fluorescent intensity of newborn heterozygous and homozygous RCLG transgenic mice (3-day-old) selected from Figure 6F; **H.** *Ex vivo* fluorescence imaging of mouse tail tip from adult offspring (3-week-old) mentioned in Figure 6F. **I–M.** Verifying the visually-identified homozygous RCLG transgenic mice by mouse mating. The homozygous RCLG transgenic mice [i.e., 1235(♂), 1248(♂), 1249(♂), 1308(♂) and 1341(♀)] determined by this optical method were further confirmed by mouse mating (see Supplementary Table S3 for details). Figure 6I, J, K, L demonstrate the representative embryo red fluorescence imaging [under stereo fluorescence microscope (Nikon, AZ100)] for blastocysts (3.5 days) obtained from female FVB/N mice which had been mated to male RCLG transgenic mice, such as 1235(♂), 1248(♂), 1249(♂) or 1308(♂), respectively. Figure 6M indicates the typical whole-body red fluorescence imaging for newborn offspring (3 day-old) derived from mating 1341(♀) with male FVB/N mice. The results obtained by embryo or whole-body fluorescence imaging for blastocysts or newborn offspring derived from mating 1235(♂), 1248(♂), 1249(♂), 1308(♂) and 1341(♀) with more FVB/N mice, respectively, are summarized in Supplementary Table S3.

To confirm the practicality of *in vivo* and *ex vivo* fluorescence imaging in identifying transgenic mice homozygous for transgene(s), we further used this optical assay to determine the zygosity in other transgenic mouse strains, such as RCLG transgenic mouse strain harboring mRFP gene under the control of CAG promoter (details are presented in the figure legend of Figure 1, Figure 2 and Figure 6). Two RCLG transgenic founders (referred to as 190^#^ and 225^#^) were derived from the microinjection of RCLG transgene fragment into the pronuclear of one cell-stage (data not shown). Procedure for establishing the homozygous RCLG transgenic mouse colonies is the same as the procedure for setting up the homozygous RLG transgenic mouse colonies (Figure 3). Organ-specific fluorescence imaging of mouse ear and tail tip from the littermates (total mice: 7) derived from brother sister mating of mRFP-positive heterozygous RCLG transgenic animals [generated by mating between RCLG transgenic mouse (190^#^, ♀) and wildtype C57BL/6J mouse (♂)] showed transgene transmission to the offspring (5 mRFP-positive and 2 mRFP-negative) following expected Mendelian laws (Figure 6A, 6B). The *ex vivo* qualitative and quantitative imaging data shown in Figure 6A, 6B and Supplementary Table S2 allow us to rapidly and readily find that two mRFP-positive transgenic mice [referred to as 1248(♂) and 1249(♂)] show more strong red fluorescence (Figure 6A, 6B) and very high fluorescence intensity (FI) in mouse ears (Supplementary Table S2), compared with the rest of 3 mRFP-positive littermates. Thus, based on the fluorescence imaging data, two mRFP-positive RCLG transgenic mice [i.e., 1248(♂) and 1249(♂)] are readily determined to be homozygous for RCLG transgene, which is perfectly consistent with the determination by mouse mating (Figure 6I and Supplementary Table S3). In addition, more homozygous RCLG transgenic mice, such as 1235(♂)(Figure 6C, 6D), 1308(♂)(Figure 6E), 1341(♀)(Figure 6F–6H), 806(♂) (data not shown), 1263(♂) (data not shown), and 1270(♀)(data not shown), were visually and readily distinguished from heterozygous transgenic alleles by this optical screening approach (Figure 6C–6H and Supplementary Table S2). As expected, these results are perfectly consistent with determination by mouse mating (Figure 6J–6M and Supplementary Table S3), indicating the practicality and reliability of this optical screening method. Furthermore, the whole-body imaging (Supplementary Figure S2 and Supplementary Figure S3) illustrates ~1.8-fold discrepancies in the red fluorescence intensity between adult homozygous (i.e., 1249) and heterozygous (i.e., 1246) RCLG transgenic mice, and between adult homozygous (i.e., 1235) and heterozygous (i.e., 1240) RCLG transgenic mice.

Additionally, two Rm17LG transgenic mice homozygous for Rm17LG transgene were easily, rapidly and precisely characterized by this visual screening method (Supplementary Figure S4), which was confirmed by mouse mating (data not shown).

In summary, the above-mentioned findings fully demonstrate that *in vivo* and *ex vivo* fluorescence imaging offers a visual, simple, rapid and reliable alternative method to the traditional approaches (i.e., mouse mating) in identifying homozygous transgenic mice harboring mRFP transgene under the control of CAG promoter.

## DISCUSSION

### Reliability and repeatability of this optical method

As mentioned above in “Introduction section”, the establishment and maintenance of transgenic mouse strains require being able to rapidly and readily distinguish homozygous from heterozygous mice in most cases [15]. The reporter gene (i.e., mRFP) (Supplementary Table S1), which is regulated under the control of a ubiquitous promoter in transgenic mice, greatly facilitates us to simply, rapidly and optically identify the homozygous transgenic mice by the *in vivo* fluorescence imaging, as strongly confirmed by these findings presented in this study. Theoretically, according to Mendelian inheritance laws, the brother-sister mating of heterozygous transgenic mice (F1 generation) result in 25% homozygous transgenic, 50% heterozygous transgenic and 25% nontransgenic offspring. Following the expected Mendelian inheritance laws, the information from this optical screening method greatly facilitated us within the shortest time period to simply, rapidly and optically identify the homozygous RLG transgenic mice (10 mice), the homozygous RCLG transgenic mice (8 mice) and the homozygous Rm17LG transgenic mice (2 mice), which was further confirmed by segregation ratio analysis in the offspring of RLG, RCLG or Rm17LG transgenic mice and wild-type mouse mating. More importantly, the *in vivo* fluorescence imaging allows scientists to readily, clearly and immediately differentiate homozygous from heterozygous transgenic alleles at birth (Figure 4B, Figure 6F and Supplementary Figure S4A). In summary, the robust optical method mentioned in this study has been confirmed to be a simple, rapid, visual, reliable and immediate screening tool (Supplementary Table S4) for identifying homozygous from heterozygous transgenic mice.

### Practicality and advantages of this optical assay

A significant challenge for scientists over the next few decades is to annotate the human genome with functional information. This effort will enable us to gain a full understanding of the molecular mechanisms and pathways underlying normal development, as well as those responsible for pathogenesis. One powerful approach is the transgene overexpression of any given gene(s) in genetically engineered mice to explore the role(s) of the gene(s) *in vivo*. Currently, the conditional transgenic mice are becoming increasingly popular for precisely regulate gene expression in a temporal and/or spatial pattern [2–4, 14, 17, 18, 21–25, 28–32]. These general transgenic constructs carrying fluorescence reporter gene (Supplementary Table S1) can be widely used to readily realize both the conditional (including temporal, spatial or spatiotemporal) and the constitutive (including ubiquitous or organ/tissue/cell-specific) transgene over-expression in transgenic mice. At this moment, the above-mentioned fluorescence reporter transgene (i.e., GFP, RFP, etc) under the control of a ubiquitous promoter allows investigators to perform the *in vivo* fluorescence imaging to rapidly, optically and immediately differentiate homozygous from heterozygous transgenic animals.

At present, the real-time quantitative PCR and mouse mating are widely and frequently employed to determine the zygosity status of mice transgenic for gene(s) of interest [15]. Despite that mouse mating may seem simpler and more straightforward than real-time quantitative PCR for zygosity analysis, mating is very time-consuming. Clearly, breeding all these animals is very expensive, labor-consuming. From an ethical point of view, it seems unreasonable to breed many mice with the unique goal of characterizing the zygosity status of the progenies [15].

The real-time quantitative PCR offers a reliable and accurate alternative method to these above-mentioned approaches in “Introduction section” in identifying homozygous transgenic animals [15]. When the researchers need to characterize the homozygous transgenic mice which harbor fluorescence reporter transgene (i.e., GFP, RFP, etc) under the control of a ubiquitous promoter, Supplementary Table S4 fully demonstrates that this *in vivo* fluorescence imaging offers a technically simpler, quicker, non-invasive, visual, reliable and accurate alternative to the classical real-time quantitative PCR in identifying homozygous transgenic mice, as strongly supported by the present study.

### Principium for identifying homozygous transgenic mice by *in vivo* fluorescence imaging

**Figure 7:**
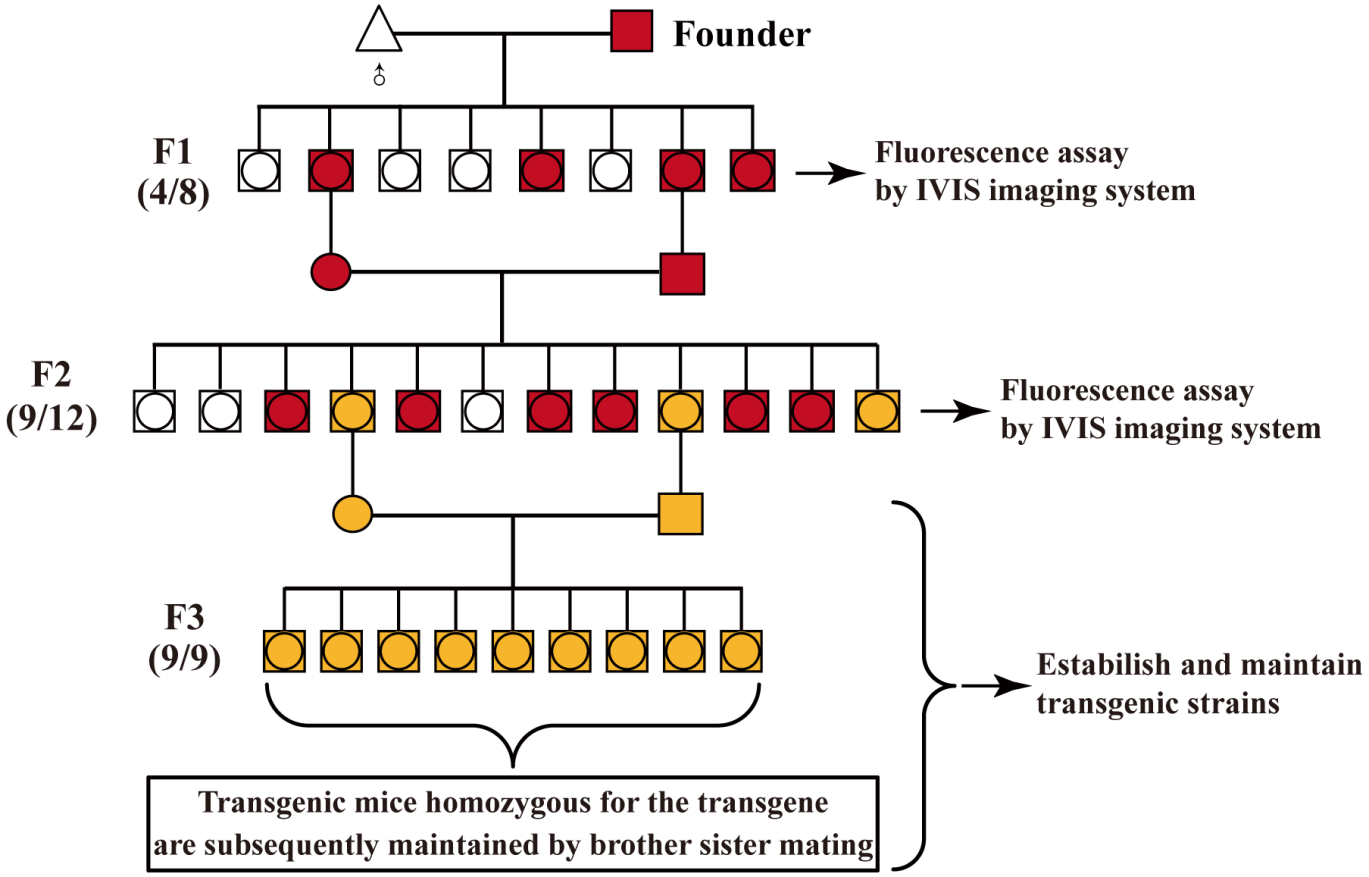
General procedure for identifying the homozygous transgenic mice by *in vivo* fluorescence imaging Founder is bred to the corresponding wild-type mouse strain. Littermates are screened for the presence of the fluorescence reporter transgene (FRT) (i.e., mRFP and EGFP) by *in vivo* fluorescence imaging. Numbers in parentheses shown on the left of map imply the ration of transgenic to non-transgenic plus transgenic offspring. 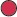
, heterozygote transgenic male; 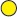, heterozygote transgenic female; 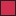
, homozygous transgenic male; 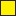, homozygous transgenic female; △, wild-type mouse; 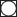
: these mRFP-negative newborn offspring are sacrificed after in vivo fluorescence imaging, so it is impossible to distinguish between male and female. 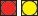
: these mRFP-positive newborn offspring are sacrificed after in vivo fluorescence imaging, so it is impossible to distinguish between male and female.

As mentioned above, this optical screening method was employed to visually identify 20 transgenic mice homozygous for three different transgenes [i.e., RLG (10), RCLG (8) and Rm17LG(2)] within the shortest time period. As shown in Figure 7, we put forward the following general protocol for characterizing homozygous transgenic mouse strains carrying the fluorescence reporter transgene (FRT) (i.e., mRFP) under the control of a ubiquitous promoter by *in vivo* fluorescence imaging.

#### 1 The production of F2-generation transgenic littermates

F2-generation littermates were produced from brother-sister mating of FRT-positive heterozygous male and female (F1 generation), which were derived from mating between FRT-positive transgenic founder (F0 generation) and the corresponding wild-type mouse strain.

#### 2 Whole-body fluorescence imaging for all of newborn F2-generation littermates

Mouse mothers who just give rise to newborn pups have been known to eat their young when threatened. For optimal visualization of fluorescence without interference from black fur, and for taking photo together for all of newborn littermates placed in a Petri dish, the whole-body fluorescence imaging for all of newborn F2-generation littermates using small animal *in-vivo* imaging system (i.e., IVIS system) had better be carried out 3 (preference) to 5 days after mouse birth. Moreover, all of newborn F2-generation siblings are recommended to be laid in a 100 mm Petri dish (i.e., Corning) when they are imaged together.

#### 3 Determination of the homozygous transgenic individual(s) based on the immediate data analysis of *in vivo* fluorescence imaging

Based on Mendelian inheritance laws, the brother-sister mating of heterozygous transgenic animals lead to 25% homozygous transgenic offspring. Generally speaking, 1 to 3 homozygous transgenic animal(s) can be found from F2-generation littermates because the mouse number of each littermate is limited and generally not above 12. Additionally, the actual number of homozygous transgenic animal(s) depends on the total number of each littermate. In this study, 1, 2 or 3 transgenic animal(s) homozygous for the specific transgene was/were found in Figure 6C, 6D, 6E, Figure 6A, 6B, 6F, Supplementary Figure S4A, or Figure 4B, respectively.

Next, we show you how to screen their offspring for the 1:4 that will be homozygous for the transgene. Firstly, the ex and *in vivo* qualitative fluorescent imaging help us fast and readily differentiate one (Figure 6C, 6D, 6E), two (Figure 6A, 6B, 6F and Supplementary Figure S4A) or three (Figure 4B) FRT-positive transgenic individual(s) with more strong fluorescence signal from the rest (with relatively weak fluorescence signal) of FRT-positive F2-generation littermates, followed by this/these FRT-positive transgenic individual(s) with more strong fluorescence signal determined to be suspected homozygous. Secondly, the immediate comparison of the fluorescent intensity of the above-mentioned suspected homozygous transgenic mice and FRT-positive transgenic individual(s) with relatively weak fluorescence signal (Supplementary Table S2) clearly indicates that in some cases, about ~ two-fold differences in FRT expression level between homozygous and heterozygous transgenic mice can be observed (Supplementary Figure S1), while in most cases, less than 2-fold differences (Figure 4C, Figure 6G and Supplementary Figure S4B) and more than 2-fold differences [26] can be often found, due to these reasons mentioned above. Finally, following Mendelian inheritance laws, this/these suspected homozygous transgenic individual(s) can be definitely determined to be true homozygote according to the data from *in vivo* qualitative and quantitative fluorescence imaging. Furthermore, to our experience, FRT-positive transgenic individual(s) with more strong fluorescence signal (for example, Supplementary Figure 4B, Figure 6A–6F and Supplementary Figure S4A), as defined by this optical screening assay, can be directly and definitely determined to be homozygote after *in vivo* or *ex vivo* fluorescence imaging.

#### 4 Establishment and maintenance of the transgenic strains

These determined transgenic mice homozygous for the transgene are subsequently maintained by brother sister mating.

In summary, the *in vivo* fluorescence imaging, as an alternative method to the traditional approaches (i.e., mouse mating and real-time quantitative PCR), allows us to visually, rapidly, reliably and accurately determinate the homozygous RLG, RCLG and Rm17LG transgenic mice. However, the reliability, repeatability and wide application of this visual screening approach to identifying homozygous transgenic mice which are derived from these general transgenic constructs carrying fluorescence reporter gene (Supplementary Table S1) remains to be fully elucidated.

## MATERIALS AND METHODS

### Mice

The wildtype C57BL/6J mice were purchased from Center of Experimental Animals, Southern Medical University. The wild-type CD1 mice were obtained from Cyagen Biosciences (Guangzhou) Inc. The homozygous EIIa-Cre transgenic mice (FVB/N-Tg(EIIa-cre)C5379Lmgd/J) [33], the homozygous Albumin-Cre transgenic mice (B6.Cg-Tg(Alb-cre)21Mgn/J) [34] and the wild-type FVB/N mice were obtained from Model Animal Research Center of Nanjing University. All animal care and experimentation were performed according to the Study and Ethical Guidelines for Animal Care, handling and termination established by the Subcommittee of Southern Medical University on laboratory animal care. The presented work was approved by the ethical committee of Southern Medical University and is covered by Chinese animal husbandary legislation.

### Production of the RLG transgenic mice

The vector of pCAG-RLG [17, 18] was generously provided by Prof. Manuela Martins-Green (University of California, Riverside, USA). As shown in Figure 2A and Figure 5A, a potent, ubiquitous CMV/β-actin promoter is used to drive a series of cassettes in the vector pCAG-RLG, including a floxed mRFP followed by three copies of a transcription-stopping polyA sequence (3 × PolyA) and a downstream internal ribosome entry site (IRES)-based bicistronic transcript, which includes open-reading frames for firefly luciferase (Luc) and EGFP. The parental vector pCAG-RLG has a built-in multiple cloning site (MCS) for cloning of any gene(s). As shown in Figure 5A, only mRFP will be transcribed and expressed properly from this construct in the absence of Cre-mediated recombination, as the expression of the Luc and EGFP transgenes is prevented by a STOP sequence flanked by *lox* P sites. When Cre-mediated recombination occurs, the floxed mRFP +3 × PolyA is excised, and the expression of Luc and EGFP transgenes will be activated.

RLG transgenic mice were generated by microinjection of single cell embryos using standard techniques as previously described [12]. The C57BL/6 mouse strain was used as the source of embryos for the micromanipulation and for subsequent breeding trials. For microinjection, the ~8.8-kb fragment of transgene RLG (Figure 2) was released free from the vector backbone of pRLG via digestion with *Ssp* I and *Sfi* I, respectively, thereafter isolated and purified using the QIA quick gel extraction kit (Qiagen, Germany), diluted to a final concentration of 2 μg/ml DNA injection buffer (10 mM Tris/0.1 mM EDTA, pH 7.4), and then microinjected into the pronuclear of one cell-stage fertilized embryos. About 20~30 DNA-injected fertilized eggs were implanted into the oviducts of one recipient pseudopregnant ICR mouse, and developed to term. Subsequently, we preliminarily screened RLG transgenic mice from potential transgenic founders by mRFP assay using the IVIS Lumina Imaging System (Referred to as IVIS system) from Xenogen (Xenogen Corp., Alameda, CA) 2–3 days after birth, followed by confirming the results of mRFP assay by PCR-based genotyping.

### Production of the RCLG transgenic mice

The human cripto-1 cDNA was cloned into the multiple cloning site (MCS) of the parental vector pCAG-RLG (mentioned in Figure 2A) to generate RCLG transgenic construct. To generate RCLG transgenic mice, the purified fragment of transgene RCLG released free from the vector backbone of pCAG-RCLG via the digestion with *Ssp* I and *Sfi* I was microinjected into the pronuclear of one cell-stage fertilized embryos [FVB/N mouse (♀) × FVB/N mouse (♂)] according to standard techniques as previously described [12]. Protocols for producing and identifying RCLG transgenic mice were the same as the protocols for producing and identifying RLG transgenic mice described above.

### Whole animal (*in vivo*) and organ (*ex vivo*) fluorescence imaging

As mouse black fur could severely interference the optimal visualization of fluorescence, in this study the juvenile mice (3–5 days old), but no adult mice were chosen to be placed in the IVIS system and analyzed for fluorescence, as described previously [35, 36]. For organ (*ex vivo*) imaging, fresh mouse organs (i.e., ear or tail) were placed on 10 cm plates and analyzed for fluorescence using the IVIS system, as described previously [35, 36]. Data was collected as photons/sec/cm^2^ using living image software v2.50 (Xenogen).

### Genotype analysis by PCR

PCR was performed on tail genomic DNA to further identify which mice have RLG integrated into their genome. The sequences of the forward primer (FP) and reverse primer (RP) (see Figure 2 for their positions) used to amplify a 339-bp fragment of the RLG transgene were: 5′-GGGAGCGCGTGATGAAC-3′ (FP) and 5′-CGTTGTGGGAGGTGATGTC-3′ (RP). PCR conditions were as follows: pre-denaturation at 94°C for 7 min, followed by 30 amplification cycles of denaturation at 94°C for 1 min, primer annealing at 54°C for 1 min, and extension at 72°C for 30 s, and finally an additional extension at 72°C for 10 min. RLG construct DNA was used as the positive control for each PCR reaction, and genomic DNA from wildtype mice was employed as a negative control for each PCR test.

### Establishment of the homozygous RLG transgenic mouse colonies

Procedure for establishing the homozygous RLG transgenic mouse colonies was detailedly illustrated in Figure 3. Briefly, at 6–8 wk of age, the transgenic founder (i.e., J068) shown to be transgenic for RLG were mated with wildtype C57BL/6J mice to generate F1; next, mRFP-positive F1 animals derived from this founder were intercrossed to produce F2. The genotypes of the founder progeny were analyzed for the transgene inheritance by the mRFP assay (see above for details).

### Activation of firefly luciferase (Luc) expression by EIIa-Cre and Alb-Cre

The potential homozygous RLG mouse (i.e., J291) was crossed to the homozygous EIIa-Cre mouse or the homozygous Alb-Cre mouse to generate RLG/EIIa-Cre or RLG/Alb-Cre double transgenic mice, respectively, in which Luc expression was activated in a diffuse or liver-restricted pattern, as determined by the bioluminescent imaging. The bioluminescence imaging was measured using the IVIS system, as described previously [35–38].

## SUPPLEMENTARY FIGURES AND TABLES



## References

[1] Hofker MH, Deursen JV (2002). Transgenic Mouse Methods and Protocols (Methods in Molecular Biology).

[2] Bockamp E, Maringer M, Spangenberg C (2002). Of mice and models: improved animal models for biomedical research. Physiol Genomics.

[3] Lewandoski M (2001). Conditional control of gene expression in the mouse. Nat Rev Genet.

[4] Vander Weyden L, Adams DJ, Bradley A (2002). Tools for targeted manipulation of the mouse genome. Physiol Genomics.

[5] Amin R, Marfak A, Pangault C, Oblet C, Chanut A, Tarte K, Denizot Y, Cogne M (2014). The class-specific BCR tonic signal modulates lymphomagenesis in a c-myc deregulation transgenic model. Oncotarget.

[6] Ianzano ML, Croci S, Nicoletti G, Palladini A, Landuzzi L, Grosso V, Ranieri D, Dall'Ora M, Santeramo I, Urbini M, De Giovanni C, Lollini PL, Nanni P (2014). Tumor suppressor genes promote rhabdomyosarcoma progression in p53 heterozygous, HER-2/neu transgenic mice. Oncotarget.

[7] Schneeberger VE, Renm Y, Luetteke N, Huang Q, Chen L, Lawrence HR, Lawrence NJ, Haura EB, Koomen JM, Coppola D, Wu J (2015). Inhibition of Shp2 suppresses mutant EGFR-induced lung tumors in transgenic mouse model of lung adenocarcinoma. Oncotarget.

[8] Shin DH, Park JH, Lee JY, Won HY, Jang KS, Min KW, Jang SH, Woo JK, Oh SH, Kong G (2015). Overexpression of Id1 in transgenic mice promotes mammary basal stem cell activity and breast tumorigenesis. Oncotarget.

[9] Tung YT, Huang PW, Chou YC, Lai CW, Wang HP, Ho HC, Yen CC, Tu CY, Tsai TC, Yeh DC, Wang JL, Chong KY, Chen CM (2015). Lung tumorigenesis induced by human vascular endothelial growth factor (hVEGF)-A165 overexpression in transgenic mice and amelioration of tumor formation by miR-16. Oncotarget.

[10] Yuan J, Jiang B, Zhang A, Qian Y, Tan H, Gao J, Shao C, Gong Yl (2015). Accelerated hepatocellular carcinoma development in CUL4B transgenic mice. Oncotarget.

[11] Wang F, Shen X, Li S, Chen L, Wang Y, Qin J, Zhou G, Peng Y, Feng X, Li R, Liang C (2015). Splenocytes derived from young WT mice prevent AD progression in APPswe/PSENldE9 transgenic mice. Oncotarget.

[12] Nagy A, Gertsensten M, Vintersten K, Behringer R (2003). Manipulating the Mouse Embryo: A Laboratory Manual.

[13] Singer O, Tiscornia G, Ikawa M, Verma IM (2006). Rapid generation of knockdown transgenic mice by silencing lentiviral vectors. Nat Protoc.

[14] Lois C, Hong EJ, Pease S, Brown EJ, Baltimore D (2002). Germline transmission and tissue-specific expression of transgenes delivered by lentiviral vectors. Science.

[15] Tesson L, Rémy S, Ménoret S, Usal C, Anegon I (2010). Analysis by quantitative PCR of zygosity in genetically modified organisms. Methods Mol Biol.

[16] Hoffman RM (2008). Recent advances on *in vivo* imaging with fluorescent proteins. Methods Cell Biol.

[17] Zheng L, Njauw CN, Martins-Green M (2008). A one-plasmid conditional color-switching transgenic system for multimodal bioimaging. Transgenic Res.

[18] Zheng L, Njauw CN, Martins-Green M (2007). A hCXCR1 transgenic mouse model containing a conditional color-switching system for imaging of hCXCL8/IL-8 functions *in vivo*. J Leukoc Biol.

[19] Vintersten K, Monetti C, Gertsenstein M (2004). Mouse in red: red fluorescent protein expression in mouse ES cells, embryos, and adult animals. Genesis.

[20] Hadjantonakis AK, Macmaster S, Nagy A (2002). Nagy, Embryonic stem cells and mice expressing different GFP variants for multiple non-invasive reporter usage within a single animal. BMC Biotechnol.

[21] Amendola M, Venneri MA, Biffi A, Vigna E, Naldini L (2005). Coordinate dual-gene transgenesis by lentiviral vectors carrying synthetic bidirectional promoters. Nat Biotechnol.

[22] Marumoto T, Tashiro A, Friedmann-Morvinski Dc (2009). Development of a novel mouse glioma model using lentiviral vectors. Nat Med.

[23] Qiu L, Wang H, Xia X, Zhou H, Xu Z (2008). A construct with fluorescent indicators for conditional expression of miRNA. BMC Biotechnol.

[24] Szulc J, Wiznerowicz M, Sauvain MO, Trono D, Aebischer Pc (2006). A versatile tool for conditional gene expression and knockdown. Nat Methods.

[25] Welm BE, Dijkgraaf GJ, Bledau AS, Welm AL, Werb Z (2008). Lentiviral transduction of mammary stem cells for analysis of gene function during development and cancer. Cell Stem Cell.

[26] Cao YA, Bachmann MH, Beilhack A (2005). Molecular imaging using labeled donor tissues reveals patterns of engraftment, rejection, and survival in transplantation. Transplantation.

[27] Zhu H, Wang G, Li G (2005). Ubiquitous expression of mRFP1 in transgenic mice. Genesis.

[28] Bornkamm GW, Berens C, Kuklik-Roos Cl (2005). Stringent doxycycline-dependent control of gene activities using an episomal one-vector system. Nucleic Acids Res.

[29] Kleinhammer A, Deussing J, Wurst W, Kühn R (2011). Conditional RNAi in mice. Methods.

[30] Ryding ADS, Sharp MGF, Mullins JJ (2001). Conditional transgenic technologies. J Endocrinol.

[31] Sun Y, Chen X, Xiao D (2007). Tetracycline-inducible expression systems: new strategies and practices in the transgenic mouse modeling. Acta Biochim Biophys Sin (Shanghai).

[32] Wang X (2009). Cre transgenic mouse lines. Methods Mol Biol.

[33] Lakso M, Pichel JG, Gorman JR, Sauer B, Okamoto Y, Lee E, Alt FW, Westphal H (1996). Efficient *in vivo* manipulation of mouse genomic sequences at the zygote stage. Proc Natl Acad Sci U S A.

[34] Postic C, Shiota M, Niswender KD, Jetton TL, Chen Y, Moates JM, Shelton KD, Lindner J, Cherrington AD, Magnuson MA (1999). Dual roles for glucokinase in glucose homeostasis as determined by liver and pancreatic beta cell-specific gene knock-outs using Cre recombinase. J Biol Chem.

[35] Du T, Jia J, Lin X, Xie R, Li J, Xiao D, Xu K (2014). Generation of Rm21LG transgenic mice: a powerful tool to generate conditional overexpression of miR-21 that is involved in oncogenesis. Biotechnol Lett.

[36] Lin X, Jia J, Du T, Li W, Wang X, Wei J, Lin X, Zeng H, Yao L, Chen X, Zhuang J, Weng J, Liu Y (2015). Overexpression of miR-155 in the liver of transgenic mice alters the expression profiling of hepatic genes associated with lipid metabolism. PLoS One.

[37] Rong XX, Wei F, Lin XL, Qin YJ, Chen L, Wang HY, Shen HF, Jia LT, Xie RY, Lin TY, Hao WC, Yang J, Yang S, Chen YS, Huang WH, Li AM, Sun Y, Luo RC, Xiao D (2015). Recognition and killing of cancer stem-like cell population in hepatocellular carcinoma cells by cytokine-induced killer cells via NKG2d-ligands recognition. Oncoimmunology.

[38] Wei F, Rong XX, Xie RY, Jia LT, Wang HY, Qin YJ, Chen L, Shen HF, Lin XL, Yang J, Yang S, Hao WC, Chen Y, Xiao SJ, Zhou HR, Lin TY, Chen YS, Sun Y, Yao KT, Xiao D (2015). Cytokine-induced killer cells efficiently kill stem-like cancer cells of nasopharyngeal carcinoma via the NKG2D-ligands recognition. Oncotarget.

