# Supplementary material for: Simple and rapid determination of homozygous transgenic mice via in vivo fluorescence imaging
Source: Oncotarget. 2015 Oct 12;6(36):39073–87. doi: 10.18632/oncotarget.5535 (PMC4766372; doi:10.18632/oncotarget.5535)
Supplement: Supplementary file 1 [file oncotarget-06-39073-s001.pdf]

SUPPLEMENTARY FIGURES AND TABLES

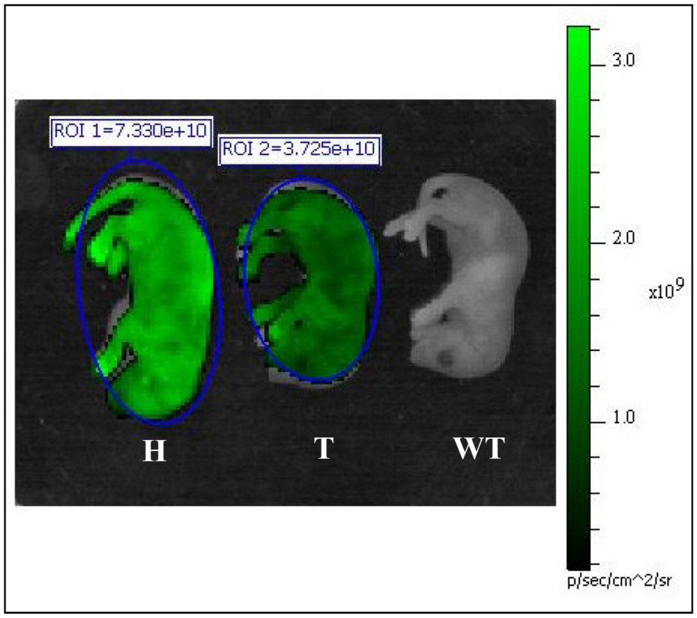

Supplementary Figure S1: Fluorescent intensity of newborn heterozygous (T) and homozygous (H) EGFP transgenic mice. The homozygous EGFP transgenic mouse line with an "enhanced" GFP (EGFP) cDNA under the control of a chicken beta-actin promoter and cytomegalovirus enhancer (CAG) makes all of the tissues appear green under excitation light. Homozygous EGFP transgenic mice were produced by mating homozygous male and female, while heterozygous EGFP transgenic mice were generated by mating homozygous male with wildtype C57BL/6J female. Fluorescent intensity (photons/sec/cm2) of newborn heterozygous (T) and homozygous (H) EGFP transgenic mice (3 days old) was measured by the IVIS Lumina Imaging System (referred to as IVIS System) from Xenogen. Abbreviation: WT: wild-type C57BL/6J mice.

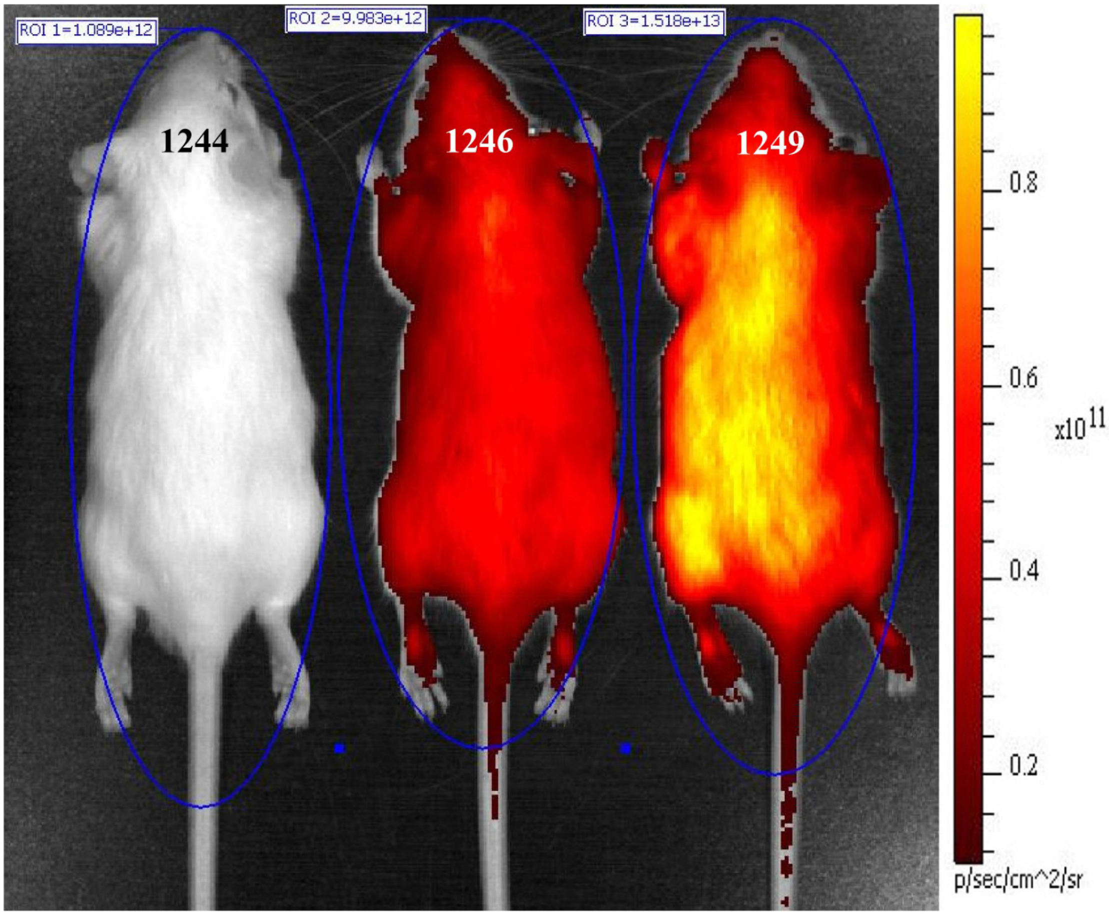

Supplementary Figure S2: Fluorescent intensity of adult heterozygous (1246) and homozygous (1249) RCLG transgenic mice. Fluorescent intensity of adult homozygous mouse (1249), heterozygous mouse (1246) and mRFP-negative mouse (1244) selected from adult offspring derived from mating between RCLG transgenic mouse (590, ♀) and RCLG transgenic mouse (592, ♂) was determined by IVIS System. The fluorescence imaging pictures of mouse ears and tail tips from adult offspring derived from mating between 590 and 592 was indicated in Figure 6A, B. Other details as in Figure 6 and Supplementary Table S2.

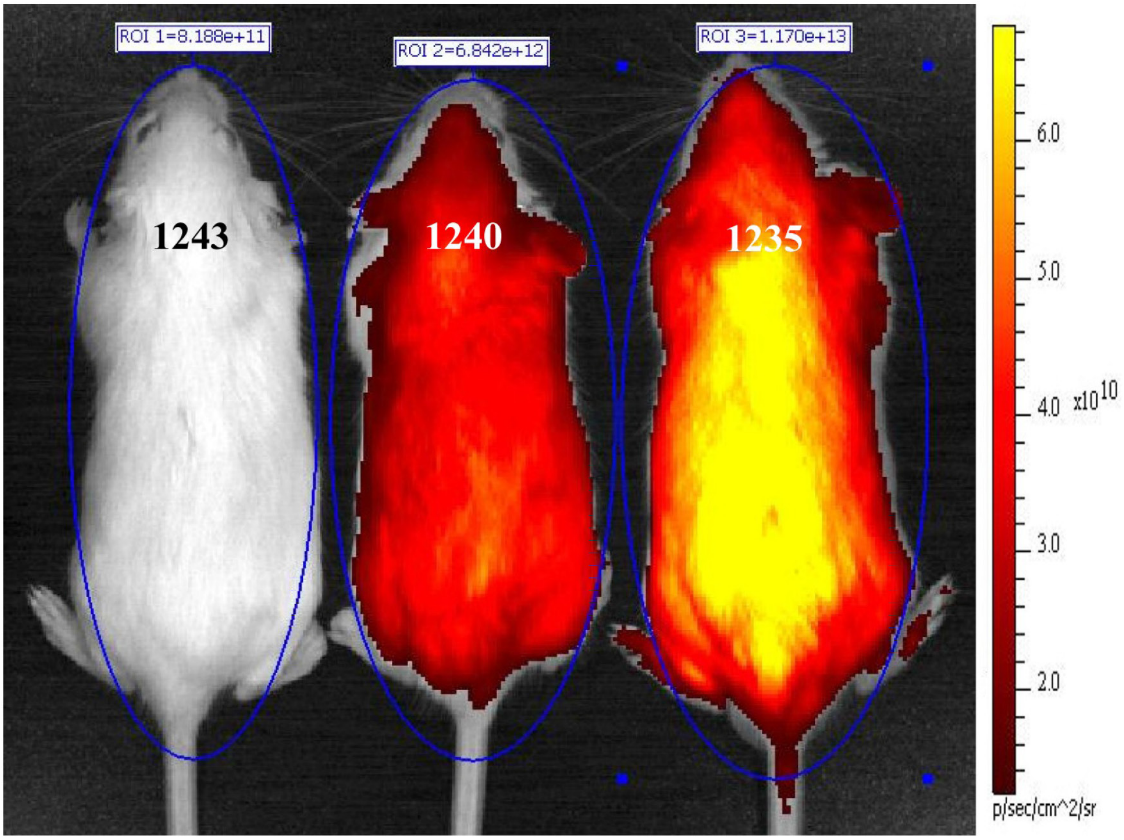

Supplementary Figure S3: Fluorescent intensity of adult heterozygous (1240) and homozygous (1235) RCLG transgenic mice. Whole-body animal red fluorescence imaging of adult homozygous mouse (1235), heterozygous mouse (1240) and mRFP-negative mouse (1243) selected from adult offspring derived from mating between RCLG transgenic mouse (589, ♀) and RCLG transgenic mouse (596, ♂) was performed by IVIS System. The fluorescence imaging pictures of mouse ears and tail tips from adult offspring derived from mating between 589 and 596 were indicated in Figure 6C, 6D. Other details as in Figure 6 and Supplementary Table S2.

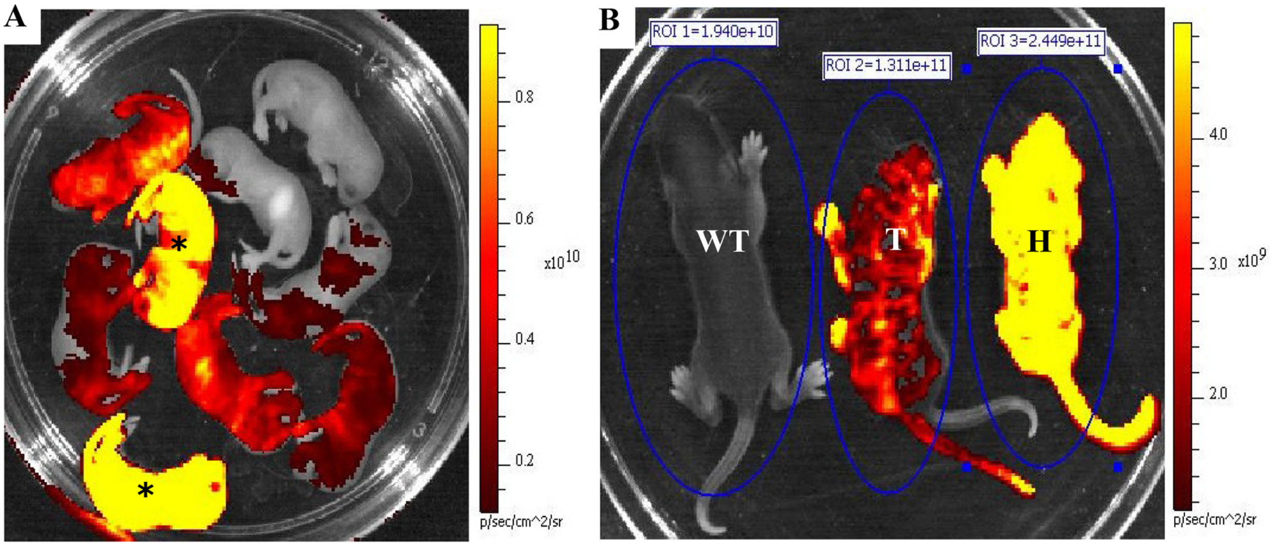

Supplementary Figure S4: Rapidly and readily screening homozygous Rm17LG transgenic mice by *in vivo* qualitative and quantitative fluorescence imaging. The human miR-17–92 was cloned into the multiple cloning site (MCS) of the parental vector pCAG-RLG (mentioned in Figure 2A) to generate CAG-Rm17LG transgenic construct used for producing Rm17LG transgenic mice. Protocols for producing and identifying Rm17LG transgenic mice were the same as the protocols for producing and identifying RLG transgenic mice included in "MATERIALS AND METHODS" section. Two Rm17LG transgenic founders (i.e., 286 and 551) were attained. mRFP expression of whole body was assayed by IVIS System. **A.** Whole-body fluorescence imaging of newborn offspring (3-day-old) produced by mating between heterozygous Rm17LG transgenic mouse (556, ♀) and Rm17LG transgenic mouse (553, ♂) derived from by intercrosses of founder 286 and wildtype C57BL/6J mouse (♀). Homozygous Rm17LG transgenic mice, as defined by *in vivo* qualitative fluorescence imaging, were marked by asterisk (*). **B.** Fluorescent intensity of heterozygous (T) and homozygous (H) Rm17LG transgenic mice (8-day-old).

Supplementary Table S1: Summary of general transgenic constructs (used to generate transgenic mice) developed by the various labs

Plasmid name	Reportergene	Promoter forreporter gene	Function description of constructs	References
pRLG3A	mRFP	CAG	Transgenic (Tg) mice developed from this construct can offer:<br>1. Cre/lox P-mediated spatial and/or temporal target transgene expression by various Cre Tg mice;<br>2. mRFP facilitates the visual identification of Tg mice	[1, 2]
pCAG-EGFP/ RFP-miRNAint	EGFP	CAG	Tg mice developed from this construct can offer:<br>1. Cre/lox P-mediated spatial and/or temporal microRNA expression by various Cre Tg mice;<br>2. EGFP facilitates the visual identification of Tg mice	[3]
pTomo	mRFP	CMV	Tg mice developed from this construct can offer:<br>1. Cre/lox P-mediated spatial and/or temporal target transgene expression by various Cre Tg mice;<br>2. mRFP facilitates the visual identification of Tg mice	[4]

(*Continued*)

Plasmid name	Reportergene	Promoter forreporter gene	Function description of constructs	References
Bidirectional lentiviral vectors	EGFP	CMV<br>hUb-C<br>or<br>PGK	Lentiviral vectors could carry synthetic bi-directional promoters (i.e., ubiquitous and/or tissue/cell- specific promoter) that mediate coordinate transcription of two mRNAs in a ubiquitous or a tissue-specific manner;<br>Tg mice developed from these constructs can offer:<br>1. the constitutive expression of reporter gene under the control of a ubiquitous promoter;<br>2. the ubiquitous or tissue-specific expression of target gene(s) under the control of a ubiquitous or a tissue/ cell-specific promoter, respectively;<br>3. EGFP facilitates the visual identification of Tg mice	[5]
pFUGW	EGFP	hUb-C	Tg mice developed from this construct can offer:<br>1. the consistent expression of reporter and target gene(s) under the control of a ubiquitous promoter;<br>2. EGFP, ZsGreen or mRFP facilitates the visual identification of Tg mice	[6]
pEF1a-IRES-ZsGreen	ZsGreen	EF-1α		Dr. Jeng-shin Lee's lab
pHIV-H2BmRFP	mRFP	EF-1α		[7]
A doxycycline-controllable single lentiviral vector system	EGFP	hUb-C<br>CAG<br>PGK<br>Or EF-1α	Tg mice developed from this construct can offer:<br>1. the drug-controllable expression of shRNA under the control of a human H1 promoter, followed by temporal RNA interference;<br>2. the drug-controllable expression of EGFP gene under the control of a ubiquitous promoter;<br>3. EGFP facilitates the visual identification of Tg mice	[8]
A doxycycline-controllable episomal one-vector system	EGFP	CAG	Tg mice developed from this construct can offer:<br>1. drug-controllable expression of target gene(s) (including EGFP) under control of a ubiquitous promoter;<br>2. EGFP facilitates the visual identification of Tg mice	[9]

pRLG3A, pCAG-EGFP/RFP-miRNAint belong to non-virus vectors, while pTomo, pFUGW, pEF1a-IRES-ZsGreen and pHIV-H2BmRFP belong to lentivirus vectors. Abbreviation in this table: mRFP: monomeric red fluorescent protein; EGFP: enhanced green fluorescent protein; EF-1α: human elongation factor-1 alpha.

Supplementary Table S2: Fluorescence intensity (FI) of mouse ears and/or tail tips of adult offspring produced by intercrosses of heterozygous transgenic mice

Breeding pair	Fluorescence intensity (FI) (photons/sec/cm2)												
164 (♀) × 162 (♂) (RLG mice)	ID No.	287	288*	289	290	291*	292	293	294	295*	296#	297#	298#
	ear	1.363×10^{10}	3.039×10^{10}	1.938×10^{10}	2.277×10^{10}	3.061×10^{10}	2.034×10^{10}	1.657×10^{10}	1.393×10^{10}	3.503×10^{10}	2.644×10^{9}	2.691×10^{9}	2.656×10^{9}
590 (♀) × 592 (♂) (RCLG mice)	ID No.	1244#	1245#	1246	1247	1248*	1249*	1250	WT				
	ear	6.519×10^{9}	5.848×10^{9}	6.403×10^{10}	6.881×10^{10}	9.547×10^{10}	1.058×10^{11}	4.933×10^{10}	6.079×10^{9}				
	tail	1.366×10^{9}	1.653×10^{9}	5.120×10^{9}	8.272×10^{9}	1.005×10^{10}	9.766×10^{9}	4.257×10^{9}	1.597×10^{9}				
589(♀) × 596(♂) (RCLG mice)	ID No.	1234	1235*	1236	1237	1238	1239	1240	1241#	1242#	1243#		
	ear	1.534×10^{10}	2.926×10^{10}	1.389×10^{10}	1.540×10^{10}	1.671×10^{10}	1.328×10^{10}	1.176×10^{10}	1.414×10^{9}	1.592×10^{9}	1.386×10^{9}		
	tail	7.488×10^{9}	1.112×10^{10}	7.107×10^{9}	4.669×10^{9}	4.916×10^{9}	5.063×10^{9}	6.339×10^{9}	1.225×10^{9}	1.402×10^{9}	1.641×10^{9}		
587(♀) × 596 (♂) (RCLG mice)	ID No.	1308*	1309	1310	1311	1312#							
	ear	2.813×10^{10}	1.041×10^{10}	1.115×10^{10}	1.671×10^{10}	9.733×10^{8}							
	tail	3.477×10^{10}	1.708×10^{10}	1.206×10^{10}	1.135×10^{10}	1.526×10^{9}							
588 (♀) × 592 (♂) (RCLG mice)	ID No.	1333	1334	1335	1336	1337	1338	1339	1340	1341*	1342#		
	tail	1.422×10^{10}	2.002×10^{10}	1.304×10^{10}	1.346×10^{10}	2.072×10^{10}	1.048×10^{10}	1.782×10^{10}	1.548×10^{10}	2.496×10^{10}	2.613×10^{9}		

Fluorescent intensity of mouse ears and/or tail tips of adult offspring produced by mating of the heterozygous RLG and RCLG transgenic mice were measured by IVIS System. Homozygous RLG and RCLG transgenic mice, as defined by this optical screening approach, were marked by asterisk (*), while the mRFP-negative offspring obtained by heterozygous RLG or RCLG transgenic mouse intercrosses were marked by pound sign (#). The fluorescence imaging pictures of mouse ears or/and tail tips from adult offspring derived from intercrosses of heterozygous RLG or RCLG transgenic mice were indicated in Figure 4 and Figure 6. Other details as in Figure 4 and Figure 6. Abbreviation: WT: wild-type mice; ID No.: identity number of offspring.

Supplementary Table S3: Summary for mating between RLG or RCLG mice and their corresponding wild-type strains

Parents		Number of offspring (O) or blastocyst (B)	Number of offspring or blastocyst	
Mother ID	Father ID		mRFP-positive	mRFP-negative
291 / RLG	C57BL/6J	6	6	0
291 / RLG	C57BL/6J	10	10	0
291 / RLG	C57BL/6J	7#	7	0
291 / RLG	C57BL/6J	8	8	0
		31 (O)	31	0
295 / RLG	C57BL/6J	9	9	0
295 / RLG	C57BL/6J	5#	5	0
295 / RLG	C57BL/6J	7	7	0
295 / RLG	C57BL/6J	8	8	0
		29 (O)	29	0
C57BL/6J	288 / RLG	9	9	0
C57BL/6J	288 / RLG	6	6	0
C57BL/6J	288 / RLG	9#	9	0
C57BL/6J	288 / RLG	7	7	
		31 (O)	31	0
287 / RLG	C57BL/6J	5# (O)	3	2
293 / RLG	C57BL/6J	6# (O)	3	3
FVB	1235 / RCLG	11#	11	0
FVB	1235 / RCLG	9	9	0
		20 (B)	20	0
FVB	1248 / RCLG	16#	16	0
FVB	1248 / RCLG	10	6	0
		26 (B)	26	0
FVB	1249 / RCLG	12#	12	0
FVB	1249 / RCLG	14	14	0
		26 (B)	26	0
FVB	1308 / RCLG	10# (B)	10	0
FVB	1308 / RCLG	10 (O)	10	0
		20	20	0
1341 / RCLG	FVB	8#	8	0
1341 / RCLG	FVB	10	10	0
		18 (O)	18	0

By *ex vivo* and *in vivo* fluorescence imaging, RLG transgenic mice (i.e., J288, J291 and J295) and RCLG transgenic mice (i.e., 1235, 1248, 1249 and 1341) were determined to be homozygous, while RLG transgenic mice (i.e., J287 and J293) were determined to be heterozygous. The representative pictures of red fluorescence imaging for newborn offspring and mouse blastocysts [marked with pound sign (#)] were shown in Figure 4E–4I and Figure 6I–6M, respectively. Other details as in Figure 4E–4I and Figure 6I–6M.

Supplementary Table S4: Comparative advantages of *in vivo* fluorescence imaging and real-time quantitative PCR in identifying homozygous transgenic mice (HTM)

Item	Fluorescence imaging	Real-time quantitative PCR
HTM determining time	Immediately after birth	4–5 weeks after birth
Visual feature	Visual	No visual
Invasive feature	Non-invasive	Invasive
Complexity	Simple	Complicated (i.e, complicated calculations)
Cut tail or not	Not	Need
Isolate tail genomic DNA or not	Not	Need
Spending time	Not time-consuming	Time-consuming
Labor intensity	Not labor-consuming	Labor-consuming
Key equipment	Small animal *in-vivo* imaging system	Fluorescence quantitative PCR instrument

REFERENCES

1. Zheng L, Njauw CN, Martins-Green M. A one-plasmid conditional color-switching transgenic system for multimodal bioimaging. Transgenic Res. 2008; 17:741–747
2. Zheng L, Njauw CN, Martins-Green M. A hCXCR1 transgenic mouse model containing a conditional color-switching system for imaging of hCXCL8/IL-8 functions *in vivo*. J Leukoc Biol. 2007; 82:1247–1256
3. Qiu L, Wang H, Xia X, Zhou H, Xu Z. A construct with fluorescent indicators for conditional expression of miRNA. BMC Biotechnol. 2008; 8:77
4. Marumoto T, Tashiro A, Friedmann-Morvinski Dc. Development of a novel mouse glioma model using lentiviral vectors. Nat Med. 2009; 15:110–116
5. Amendola M, Venneri MA, Biffi A, Vigna E, Naldini L. Coordinate dual-gene transgenesis by lentiviral vectors carrying synthetic bidirectional promoters. Nat Biotechnol. 2005; 23:108–116
6. Lois C, Hong EJ, Pease S, Brown EJ, Baltimore D. Germline transmission and tissue-specific expression of transgenes delivered by lentiviral vectors. Science. 2002; 295:868–872
7. Welm BE, Dijkgraaf GJ, Bledau AS, Welm AL, Werb Z. Lentiviral transduction of mammary stem cells for analysis of gene function during development and cancer. Cell Stem Cell. 2008; 2:90–102
8. Szulc J, Wiznerowicz M, Sauvain MO, Trono D, Aebischer Pc. A versatile tool for conditional gene expression and knockdown. Nat Methods. 2006; 3:109–116
9. Bornkamm GW, Berens C, Kuklik-Roos Cl. Stringent doxycycline-dependent control of gene activities using an episomal one-vector system. Nucleic Acids Res. 2005; 33:e137
